# Vat photopolymerization of gel polymer electrolytes with solvent-dependent performance and complex geometries for Li-ion batteries

**DOI:** 10.1038/s44172-026-00682-9

**Published:** 2026-05-05

**Authors:** Alexis Maurel, Katherine R. Gonzalez, Hugo A. Garcia, Laura C. Merrill, Ana C. Martinez

**Affiliations:** 1 Metallurgy, Materials and Biomedical Engineering Department, The University of Texas at El Paso, El Paso, TX USA; 2 Department of Aerospace and Mechanical Engineering, The University of Texas at El Paso, El Paso, TX USA; 3 Sandia National Laboratories, Albuquerque, NM USA

**Keywords:** Mechanical engineering, Batteries, Batteries

## Abstract

Additive manufacturing offers new opportunities for fabricating next-generation battery components with unprecedented control over three-dimensional architecture and spatial complexity. This study presents the development and electrochemical characterization of 3D printable gel polymer electrolytes (GPEs) based on a UV-curable PEGDA resin and a liquid electrolyte composed of 1 M LiClO_4_ in EC:DEC or EC:PC (1:1 v/v). The impact of resin-to-electrolyte ratios on ionic conductivity and processability is systematically evaluated, with 1:4 v/v identified as the optimal formulation. GPEs fabricated via vat photopolymerization exhibit high ionic conductivities of up to 3.4 × 10^-3^ S.cm^-1^ (DEC-based) and 3.1 × 10^-3^ S.cm^-1^ (PC-based), closely matching their tape-cast counterparts. Electrochemical stability is maintained up to ~4.5 V vs. Li^0^/Li^+^, with symmetric cell testing confirming effective Li^0^ plating/stripping over 100 cycles. The 3D printed GPEs retain their electrochemical performance despite performing the printing process in ambient air, demonstrating robustness and compatibility with scalable manufacturing. In addition, the GPEs can be printed into complex geometries, further underscoring their suitability for advanced device architectures. This work highlights the critical role of solvent selection and printing parameters in designing printable GPEs and paves the way toward shape-conformable, solid-state battery systems.

## Abstract (web summary)

Alexis Maurel and colleagues report 3D printable gel polymer electrolytes for Li-ion batteries using vat photopolymerization. The 3D printed materials show high ionic conductivity, stable cycling, and can be fabricated into complex geometries.

## Introduction

The growing demand for high-performance and safe energy storage systems has driven significant interest in solid and semi-solid electrolytes for lithium-based batteries. Among them, gel polymer electrolytes (GPEs) offer a promising balance between the high ionic conductivity of liquid electrolytes and the mechanical robustness of solid-state systems^1^. Composed of a polymer matrix swollen with liquid electrolyte, GPEs can suppress leakage, improving electrode–electrolyte interfacial contact, and reducing safety risks associated with dendrite formation^2^. Functional GPEs for batteries have been successfully manufactured in recent years through conventional casting and electrolyte infusion techniques^3–6^, however, traditional fabrication methods such as casting or soaking often result in GPEs with limited design flexibility, non-uniform thickness, and poor integration with complex device architectures. To address these limitations, additive manufacturing (3D printing), in particular the vat photopolymerization (VPP) process, has emerged as a promising approach for fabricating next-generation electrolytes^7–10^ due to the unprecedented spatial control over the geometry, thickness, and architecture of printed materials, enabling the creation of conformal, multi-material, and shape-customizable battery components^11,12^.

Only a limited number of studies have explored the 3D printing of GPEs via VPP. Chen et al.^13^ fabricated a zig-zag structured GPE using VPP with a poly(ethylene glycol) diacrylate (PEGDA)-based UV-curable resin incorporating 80 vol.% of a 1 M LiClO_4_ liquid electrolyte, achieving an ionic conductivity of 4.8 × 10^−3^ S cm^−1^ at room temperature. Rahman et al.^14^ employed a UV-curable resin composed of poly(vinylidene fluoride) and N,N-dimethyl acrylamide with 50 wt.% LiCl dissolved in ethylene glycol, obtaining a printed GPE with an ionic conductivity of 6.5 × 10^−4^ S cm^−1^ at room temperature. Zehbe et al.^15^ formulated an ionogel by blending 80 wt.% of lithium sulfonate-based ionic liquids with a commercial UV-curable resin, which exhibited an ionic conductivity of 0.7 × 10^−4^ S cm^−1^ at room temperature. More recently, Martinez et al.^10^ reported a VPP-printed GPE made of a 20 vol% of PEGDA-based resin and 80 vol% of 1 M NaClO_4_ in ethylene carbonate (EC):propylene carbonate (PC) 1:1 v/v + 3 wt% fluoroethylene carbonate (FEC) as electrolyte, that delivered an ionic conductivity of 3.3 × 10^−3^ S cm^−1^ at room temperature. From these limited available studies, it can be deduced that high liquid electrolyte or ionic liquid content (50–80 wt.% or vol.%) is essential to reach adequate ionic conductivity values in the 10^−3^–10^−4^ range. However, a thorough investigation into the role of solvent composition within the electrolyte remains an open challenge for future optimization.

In this context, this work addresses the lack of systematic understanding of solvent effects in 3D-printable GPEs. A PEGDA-based UV-curable resin is combined with liquid electrolytes composed of 1 M LiClO_4_ in EC:DEC or EC:PC (1:1 v/v), enabling a detailed evaluation of how resin-to-electrolyte ratios influence ionic conductivity, processability using VPP, and electrochemical stability for lithium-ion batteries. Unlike previous reports that primarily focused on achieving high liquid electrolyte content to ensure adequate ionic conductivity, this work places particular emphasis on solvent species selection as a key parameter for balancing printability and electrochemical performance. By doing so, it establishes a pathway toward the rational design of printable, shape-conformable electrolytes suited for next-generation lithium-ion battery architectures.

## Materials and methods

### Composite photo-curable GPE resins preparation

Composite GPE resins were prepared by mixing a classical UV-curable resin and liquid electrolyte in volume ratios of 1:1, 1:2, 1:3, 1:4, and 1:5. The classical polymeric resin consisted of poly(ethylene glycol) diacrylate (PEGDA, Mn 575, Sigma-Aldrich) and phenylbis(2,4,6-trimethylbenzoyl)phosphine oxide (TPO, Sigma-Aldrich) in a 1:0.005 weight ratio. Electrolytes were prepared inside an argon-filled glovebox (<0.1 ppm O_2_; <0.1 ppm H_2_O) and contained 1 M LiClO_4_ dissolved in either ethylene carbonate/diethyl carbonate (EC/DEC, 1:1 v/v, Sigma-Aldrich) or ethylene carbonate/propylene carbonate (EC/PC, 1:1 v/v, Sigma-Aldrich). The composite resins were mixed for 30 min in a closed amber-colored container and stored in a fridge outside the glovebox at 4 °C to minimize degradation. In addition, a modified PC-based composite resin was formulated by incorporating allura red (Sigma-Aldrich) as a UV-blocking additive, with a PEGDA:allura red weight ratio of 1:0.05.

### Tape cast of GPE films

Tape cast GPE films with varying resin-to-electrolyte ratios were prepared inside an argon-filled glovebox and UV-cured for 30 s using a 405 nm lamp, matching the wavelength of the VPP 3D printer employed later in this study. Circular GPE samples with a diameter of 6.35 mm (¼ in) or 15.5 mm, and thickness between 300 and 400 µm were punched from the cured films for subsequent electrochemical characterization.

### Design and printing

Computer-aided design (CAD) was carried out using nTopology (nTopology, USA). Simple disc geometries (diameter of 6.35 mm (¼ in), 10 mm, or 15.5 mm depending on the test, and a thickness of 400 µm) were created. To further illustrate the design flexibility enabled by 3D printing, a more complex geometry was generated consisting of a 25 mm × 25 mm square base with a thickness of 2 mm, incorporating a hexagonal honeycomb lattice. The lattice was constructed with three unit cells along both the *X* and *Y* directions and extruded uniformly along the *Z* axis. Moreover, to demonstrate the printability of thicker items, a 1 cm^3^ cube was designed. After generating a mesh from the implicit bodies in nTopology, the designs were exported as Standard Tessellation Language (STL) files, converting the CAD models into a series of connected triangles representing the geometries. Printing was performed using a Prusa SL1S (Czech Republic) with a layer height of 0.025 mm, without supports or a base pad. The prepared composite resins were used as material feedstock for the 3D printer. For the composite resins without allura red, disc geometries were printed with an initial layer exposure of 30 s followed by 3.5 s for subsequent layers, while the honeycomb lattice was printed with a 35 seconds initial exposure and 22.5 seconds for the remaining layers. The PC-based composite resin containing the allura red UV-blocking additive was printed using 35 s for the initial layer and 25 s for subsequent layers. All printed discs received an extra UV post-cure of 1 min inside the glovebox prior to cell assembly to minimize residual unreacted monomer.

### Optical microscopy

Optical microscopy was employed to evaluate the quality and resolution of the 3D printed geometries, particularly the fidelity of the lattice features and disc surfaces. Images were acquired using an 8-megapixel DinoLite digital microscope (Model AM8917MZT), equipped with adjustable polarization and variable magnification (up to 220x). Top-view images were collected to assess surface uniformity, infill pattern definition, and potential defects caused by overcuring or incomplete polymerization. The images were used qualitatively to compare printability across different resin formulations and geometries.

### Electrochemical characterization

For potentiostatic electrochemical impedance spectroscopy (PEIS) measurements, tape cast or 3D printed GPE discs with a diameter of 6.35 mm or 15.5 mm were sandwiched between two stainless steel plates in a coin-cell configuration. The spectra were collected over a frequency range from 2 MHz to 1 Hz, using 20 points per decade, an AC amplitude of 0.05 V, a DC offset of 0.005 V, and an AC peak value of 0.0707 V. Three independent GPE samples were prepared and measured for each composition of the DEC- and PC-based composite resins to ensure reproducibility and determine error bars. The ionic conductivity, σ (S cm^−1^), was calculated using Eq. 1, where R is the resistance (Ω), t is the thickness (cm), and A is the area (cm^2^).

1 $$\sigma =(1/R)/(t/A)$$

Linear sweep voltammetry (LSV) experiments were conducted using both tape cast GPEs (fabricated under Ar, 15.5 mm diameter) and 3D printed GPE discs (15.5 mm diameter). Each sample was positioned between a stainless-steel working electrode, and a counter and reference lithium metal disc. The liquid electrolyte was tested under the same LSV conditions, using a glass fiber (Whatman GF/D) as a separator. Measurements were performed over a voltage range of 0.01–5.0 V at a scan rate of 0.1 mV s^−1^ with an Interface 1010 potentiostat/galvanostat (Gamry Instruments, USA). Additionally, galvanostatic polarization tests were carried out on symmetric Li^0^/GPE/Li^0^ coin cells at 1 mA cm^−2^, applying one-hour charge and discharge cycles, using 15.5 mm diameter GPE samples.

## Results and discussion

The GPEs studied here consist of a lithium salt dissolved in organic solvents integrated within a photocurable polymer matrix. LiClO_4_ was selected as the lithium salt due to its low hygroscopicity (in comparison with LiPF_6_), which allows processing in air without decomposition into hydrofluoric acid or other fluorinated byproducts^16^. This design strategy minimized potential water uptake during the 3D printing step and ensured compatibility with subsequent glovebox-based battery assembly.

Two solvent systems (EC:DEC and EC:PC, 1:1 v/v) were examined alongside varying resin-to-electrolyte ratios (1:1 to 1:5 v/v) to investigate their impact on ionic conductivity, electrochemical stability, and printability in VPP. The first step involved preparing GPEs with resin-to-electrolyte ratios of 1:0, 1:2, 1:3, 1:4, and 1:5 (v/v) via tape casting, followed by UV photopolymerization in an Ar-filled glovebox. This approach was used to identify the optimal resin-to-electrolyte ratio that balanced processability and electrochemical performance before scaling up the composite resin feedstock for 3D printing. While curing conditions were selected to accomplish complete polymerization for this PEGDA system and based on a previous work^17^, the presence of trace amounts of unreacted monomer cannot be completely excluded and may contribute to electrochemical noise.

**Fig. 1:**
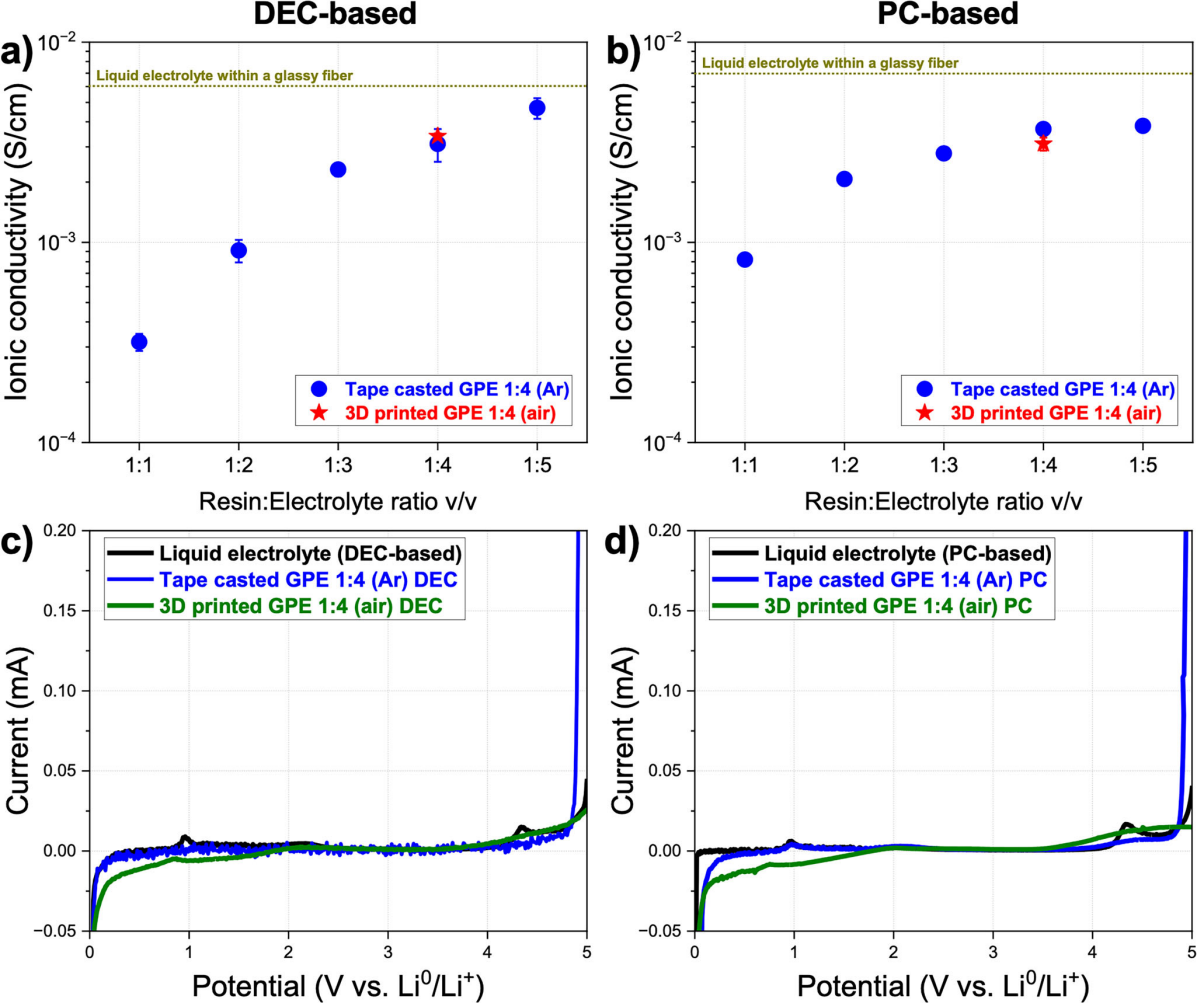
Ionic conductivity and electrochemical stability of gel polymer electrolytes (GPEs). Ionic conductivity of GPEs at different resin-to-electrolyte ratios using **a** diethyl carbonate (DEC) or **b** propylene carbonate (PC) as co-solvent. Error bars represent standard deviation (*n* = 3). Voltage window stability plots measured through linear sweep voltammetry (LSV). **c** DEC-based and **d** PC-based electrolyte.

PEIS measurements were performed on tape cast GPE films to determine the ionic conductivity of electrolytes containing DEC (Fig. 1a) or PC (Fig. 1b) as co-solvents with EC. Nyquist plots are displayed in Fig. S1. Both solvents were compared in this study due to their distinct physicochemical properties and their widespread use in liquid electrolytes for lithium-ion batteries. PC, owing to its high dielectric constant (~65 vs 2.8 for DEC), solvates Li^+^ ions more effectively than DEC. However, electrolytes containing PC, LiClO_4_, and graphite are prone to solvent co-intercalation, which can cause graphite exfoliation and poor cycling performance^18,19^. In contrast, DEC is a linear carbonate that exhibits lower viscosity and facilitates faster Li^+^ transport than PC, and promotes the formation of a more stable solid electrolyte interphase (SEI) when combined with EC^20,21^. Nevertheless, the relatively low boiling point of DEC (~127 °C vs ~241 °C for PC) raises concerns about its volatility during the 3D printing of GPEs, potentially limiting its applicability in additive manufacturing processes. The impact of the viscosity difference of DEC and PC as co-solvents may impact early-stage cross-linking kinetics, but this effect is expected to be overcome by the long curing conditions that were used in the present study.

Building on the differences in physicochemical properties of DEC and PC, the ionic conductivity of tape cast GPEs was evaluated as a function of resin-to-electrolyte ratio. As expected, conductivity increased with higher electrolyte content (lower resin proportion), consistent with trends previously observed by our group^10^. For DEC-based GPEs, the maximum conductivity reached 4.7 × 10^−3^ S cm^−1^ at a 1:5 ratio (Fig. 1a), whereas PC-based GPEs achieved 3.8 × 10^−3^ S cm^−1^ at a 1:5 ratio, closely followed by the 1:4 ratio at 3.7 × 10^−3^ S cm^−1^ (Fig. 1b). However, tape casting GPEs at the 1:4 ratio for either solvent approached the limit of handleability, and by extension, printability. Indeed, the 1:1 ratio ensured processability but yielded low conductivity (~10^−4^ S cm^−1^) due to insufficient electrolyte content. The conductivities of the 1:4 and 1:5 formulations approached those of liquid electrolytes in glass fiber (6.2 × 10^−3^ S cm^−1^ and 7.1 × 10^−3^ S cm^−1^ for DEC-based, and PC-based electrolytes, respectively), indicating that GPEs at these ratios can nearly match liquid electrolyte performance.

These observations indicate a clear tradeoff between ionic conductivity and processability: higher electrolyte content enhances conductivity but reduces mechanical handleability and printability. In this context, the rheological behavior of the composite resin becomes critical. Our group previously measured the viscosity of PEGDA-based resins at different resin-to-electrolyte ratios, specifically 1:0 (pure resin) and 1:1 v/v, as a function of temperature^10^. Given the substantial viscosity difference between PEGDA resin (~57 cP) and liquid electrolyte (~2 cP) at room temperature, it was found that incorporating electrolyte broadened the resin’s printable temperature window without significant viscosity changes. These results further suggest that electrolyte composition can influence not only ionic conductivity but also the processing characteristics of the GPEs.

Guided by the conductivity and processability trends observed from the tape cast samples, the 1:4 resin-to-electrolyte ratio was selected for scale-up and subsequent 3D printing. To further assess robustness under practical conditions, printing was performed outside the glovebox in a laboratory environment with 22% relative humidity. This approach was chosen not only because PEGDA resins are known to undergo premature polymerization under oxygen-deficient conditions^22^, but also due to the practical challenges of operating the printer inside the glovebox. The resulting ionic conductivity values are shown in Fig. 1a and b. For the DEC-based electrolyte, the 3D printed GPE achieved 3.4 × 10^−3^ S cm^−1^, which fell within the data dispersion of the tape cast samples, as indicated by the error bars. Similarly, the PC-based printed GPE reached 3.1 × 10^−3^ S cm^−1^. These results demonstrated that the printed GPEs preserved the ionic conductivity of their tape cast counterparts, confirming reproducibility across solvents and validating the selected formulation for additive manufacturing.

Both tape cast and 3D printed GPEs (1:4) were assembled into coin cells with lithium foil serving as both the reference and counter electrode to evaluate electrochemical window stability. As shown in Fig. 1c and d, all samples maintained low current up to ~4.3–4.5 V, demonstrating sufficient stability for low-voltage cathodes such as LiFePO_4_ or LiMnPO_4_. A sharp current rise was observed beyond ~4.5 V, marking the onset of oxidative decomposition^23^. Across both solvent systems (DEC-based and PC-based), the liquid electrolyte exhibited a slightly lower oxidation onset compared to the GPEs. The tape cast films exhibited high oxidative currents associated with the ether-containing segments and other functional groups present in the PEGDA backbone that can undergo oxidation at high potentials^24^. The lower oxidative currents observed for the 3D printed GPEs may be attributed to partial solvent evaporation near the surface of the samples during their transfer from the printer to the glovebox antechamber and subsequently into the glovebox. The 3D printed 1:4 GPEs remained electrochemically stable up to 4 V vs. Li^0^/Li^+^, with current levels not exceeding 0.025 mA even at 5 V. The PC-based printed GPEs behaved similarly to the DEC-based ones, though with a slightly lower baseline current between 0.5 and 2.0 V, possibly due to residual impurities or trace moisture introduced during fabrication.

**Fig. 2:**
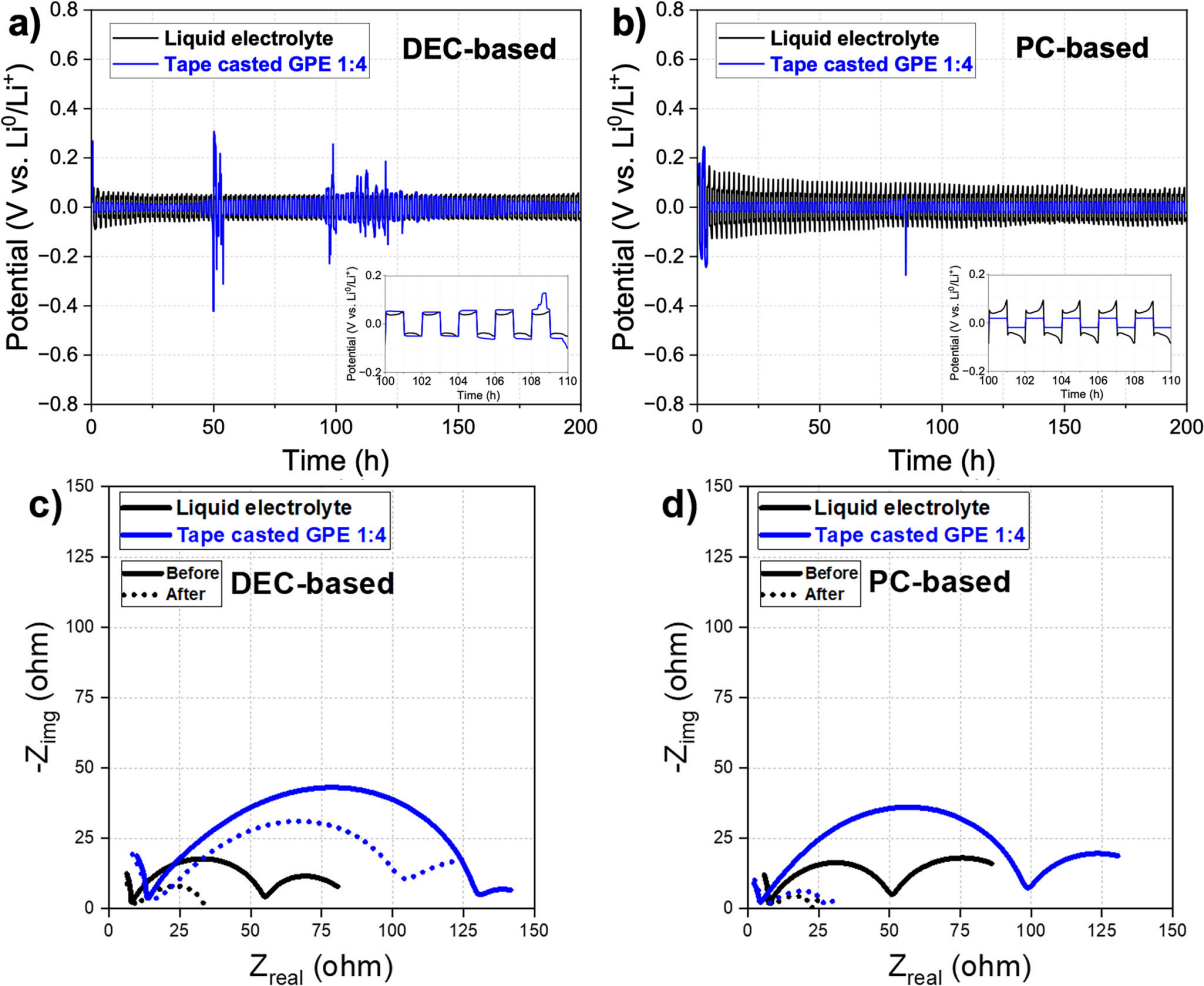
Electrochemical cycling performance and impedance response of tape-cast GPEs compared with liquid electrolytes. Galvanostatic symmetric cycling of tape cast GPE 1:4 compared with liquid electrolytes: **a** DEC-based and **b** PC-based electrolytes. The insets show an enlarged view of selected cycles. Potentiostatic electrochemical impedance spectroscopy (PEIS) of the symmetric cells with liquid electrolyte (black) and tape cast GPE (blue) using **c** DEC-based and **d** PC-based electrolytes.

To evaluate the cycling stability of GPEs in lithium-ion cells, symmetric Li^0^/tape cast GPE 1:4/Li^0^ cells were tested at a current of 1 mA cm^−2^. These cells allow monitoring of the anodic and cathodic behaviors at a single electrode–electrolyte interface without requiring a reference electrode. For comparison, liquid electrolytes in glass fiber separators were also tested (Fig. 2). For the DEC-based tests, the liquid electrolyte exhibits stable and low-amplitude voltage polarization over 200 h of cycling, indicating uniform lithium stripping and plating and a stable Li-electrolyte interface (Fig. 2a). In contrast, the DEC-based GPE shows pronounced voltage fluctuations during the initial cycling period, with spikes not exceeding 250 mV. These features are indicative of interfacial instability, likely arising from nonuniform SEI formation at the Li-GPE interfaces, heterogeneous Li^+^ transport, or incomplete wetting of the lithium surface due to the presence of the polymer matrix. With continued cycling, the voltage response of the GPE gradually stabilizes, suggesting progressive interfacial conditioning and partial stabilization of the SEI; however, the polarization remains higher and noisier than that of the liquid electrolyte throughout the test. In the PC-based tests, both the liquid electrolyte and the GPE display significantly improved cycling stability (Fig. 2b). The liquid electrolyte shows slightly higher polarization than the DEC counterpart, consistent with the higher viscosity and lower ionic conductivity of PC, yet maintains highly reproducible and symmetric voltage profiles. Notably, the PC-based GPE demonstrates stable and low-noise voltage behavior over extended cycling, closely resembling that of the liquid electrolyte. Apart from an isolated transient voltage spike, the GPE maintains consistent polarization, indicating a more stable interface. This contrast with the DEC-based GPE highlights the strong dependence of GPE interfacial performance on solvent chemistry.

PEIS was performed before and after galvanostatic cycling to complement the understanding of the observed cycling behavior. In the DEC-based tests (Fig. 2c), the liquid electrolyte exhibits two semicircles at high-medium frequencies corresponding to the SEI resistance (R_SEI_) and to the charge-transfer resistance at the Li interface (R_ct_). After cycling, both R_ct_ and R_SEI_ decreased considerably (Fig. 2c, black data). Conversely, the DEC-based tape cast GPE (Fig. 2c, blue data) shows a substantially larger R_SEI_ before cycling in comparison with the liquid electrolyte samples, which was expected due to the more complex composition including a polymer matrix. After cycling, the resistance of the bulk electrolyte (R_s_) in the tape cast GPE remained unchanged, but the R_SEI_ decreased, consistent with the stabilization of the stripping and plating after 130 h. Similarly to the DEC-based results, the PC-based liquid electrolyte exhibits a smaller R_SEI_ semicircle than its DEC-based counterpart before cycling, and displays a considerable change after cycling (Fig. 2d, black data). After cycling, both R_ct_ and R_SEI_ decreased considerably. These results indicate that the instability observed during galvanostatic cycling of the GPEs is not driven by cumulative interfacial resistance growth, but rather by dynamic interfacial heterogeneity and intermittent loss of interfacial contact^25^.

**Fig. 3:**
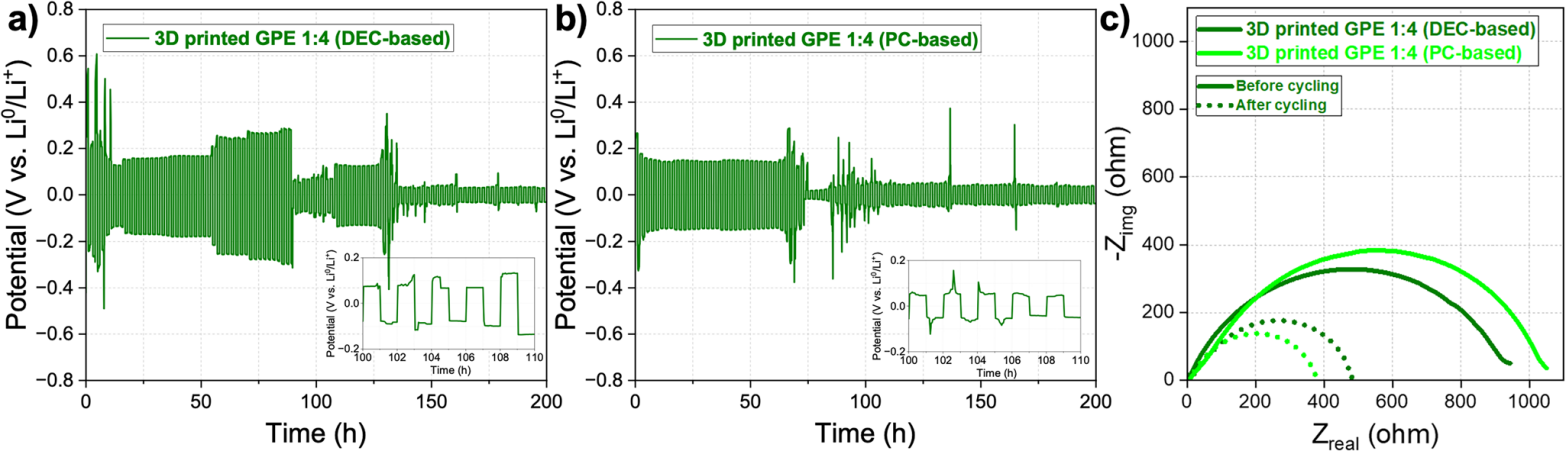
Electrochemical testing of the 3D printed GPE 1:4. Galvanostatic symmetric cycling of 3D printed GPE 1:4: **a** DEC-based and **b** PC-based electrolytes. The insets show an enlarged view of selected cycles. **c** PEIS of the symmetric cells for both 3D printed DEC-based or PC-based GPEs.

Following the evaluation of tape cast GPEs, 3D printed GPEs were examined in symmetric cells to investigate the effects of additive manufacturing and ambient-air processing on interfacial stability. Importantly, the thickness of these 3D printed GPEs was carefully tuned to match that of tape cast GPEs. Galvanostatic cycling of 3D printed GPEs in symmetric cells exhibited voltage spikes that were more pronounced during the first 20 h (10 cycles) in the DEC-based GPE cycling at 1 mA cm^2^ (Fig. 3a). This behavior was likely caused by residual moisture on the 3D printed GPEs as a consequence of printing in air. Over time, the overpotential stabilized for both GPEs, with the PC-based sample reaching steady behavior after ~130 h (65 cycles) and the DEC-based sample after ~110 h (55 cycles, Fig. 3b). In a similar manner to previous impedance results, both 3D printed GPEs exhibit lower resistance values after cycling, with the PC-based sample showing the largest decrease (Fig. 3c). In general, the impedance values of the printed samples remain higher than those of the tape cast samples, likely due to the exposure of the electrolyte to air during 3D printing, which should be mitigated if the printing is done under low H_2_O and O_2_ conditions.

**Fig. 4:**
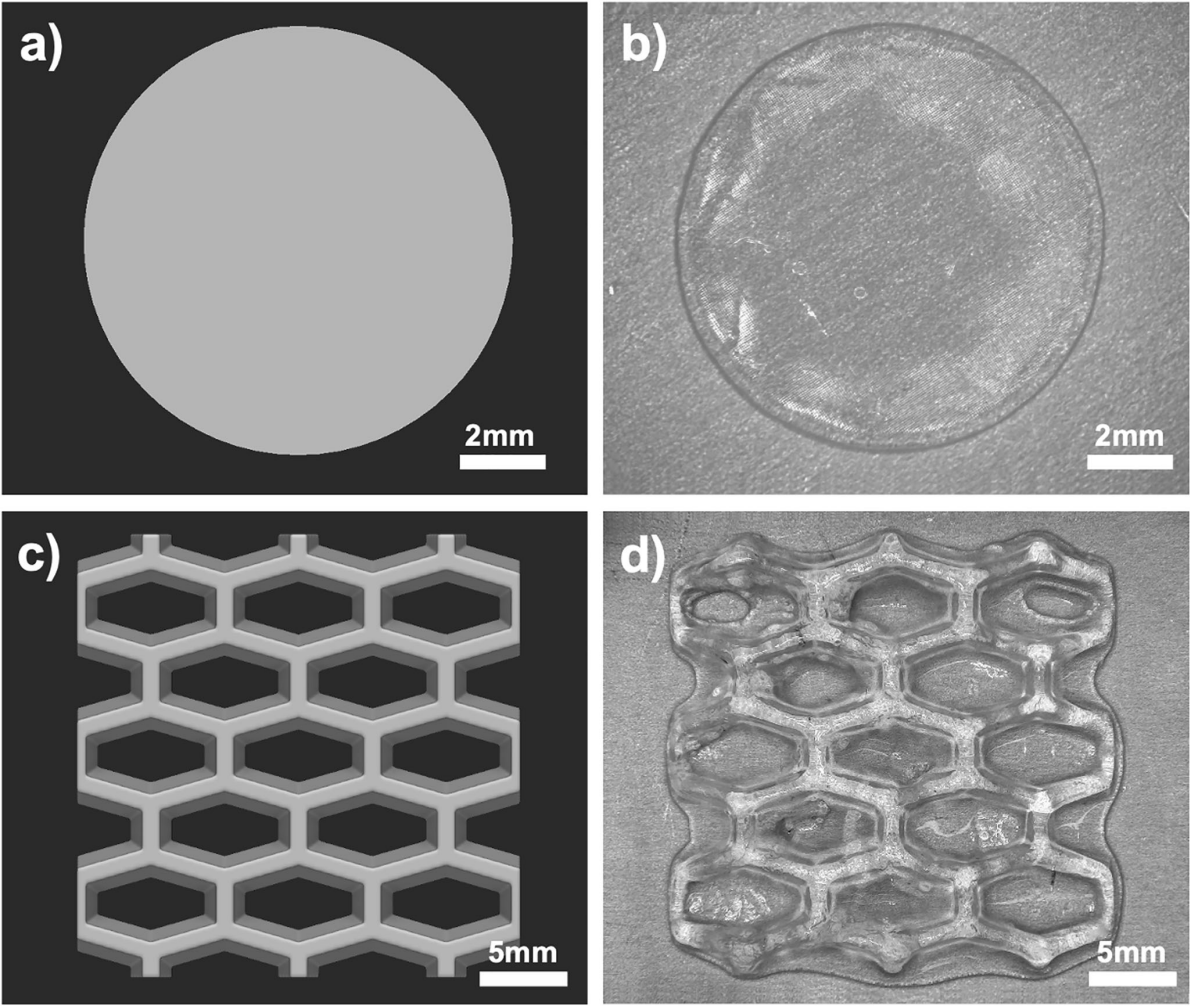
3D printed items using PC-based GPE 1:4. **a** STL model of the disc, **b** 3D printed disc, **c** STL model of the honeycomb lattice, and **d** 3D printed honeycomb structure.

Based on the physicochemical properties discussed above, and the lower interfacial resistance and better cycling charge-transfer kinetics observed during the electrochemical testing of tape cast and 3D printed GPEs, the PC-based GPE appears to be the best choice to perform printability experiments. The printability of the PC-based GPE 1:4 was evaluated using two representative geometries, a simple disc (10 mm in diameter) and a more complex honeycomb lattice with open pores (Fig. 4). The discs that were 3D printed using GPE 1:4 exhibited excellent fidelity, retaining the intended geometry and smooth surfaces, while still maintaining high ionic conductivity as shown previously. In contrast, the honeycomb lattice did not reproduce the macroporous infill pattern accurately; the transparency of the resin caused overcuring, leading to partial filling of the pores with solidified material.

**Fig. 5:**
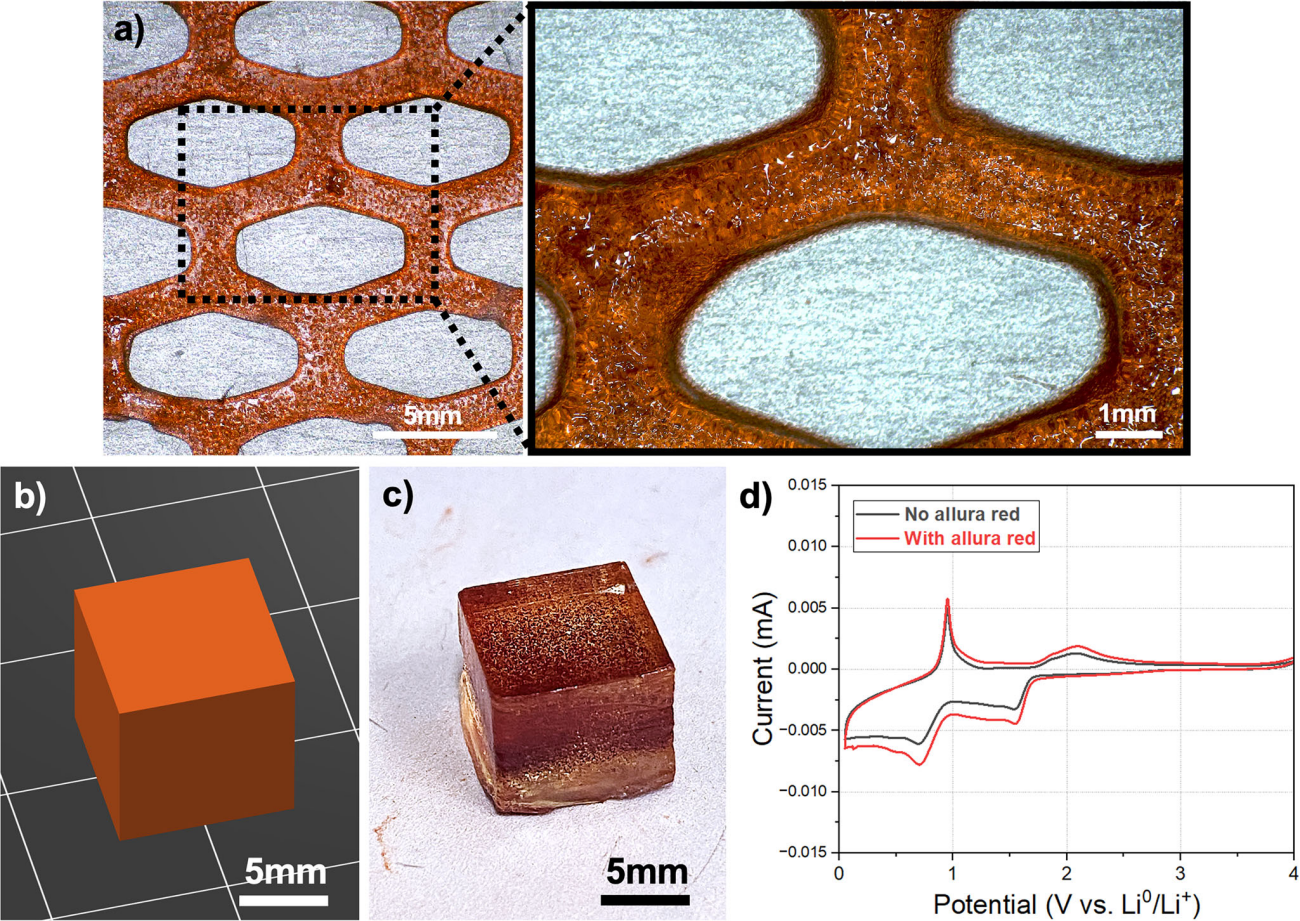
3D printing capability of PC-based GPE formulations and effect of allura red on electrochemical behavior. **a** Optical image of the 3D printed honeycomb lattice obtained from the PC-based GPE with allura red as a UV blocker. **b** STL design and **c** optical image of the 3D printed 1 cm^3^ cube obtained from the PC-based GPE with allura red as a UV blocker. **d** Cyclic voltammetry testing of half-cells containing or not allura red.

A potential approach to improve printability and prevent overcuring is the incorporation of UV blockers^26^, such as allura red, which absorb excess light and limit polymerization depth^27^. By adding allura red to the PC-based composite GPE 1:4 resin, a complex honeycomb lattice with well-defined features as well as a 1 cm^3^ cube (to demonstrate the printability of thicker items) were successfully printed (Fig. 5a–c). However, the use of UV absorbers in battery GPE must be approached with caution, as they may negatively affect electrochemical performance by affecting the SEI, solvation, and conductivity.

To the best of the authors’ knowledge, there are no published studies where allura red was intentionally incorporated to be studied as an additive, active electrochemical species, or to probe its impact on ionic transport, electrode interfaces, SEI formation, redox stability, or cycling performance. However, electrochemical oxidation and voltametric detection studies have demonstrated that allura red can undergo redox reactions in aqueous systems^28,29^. Therefore, cyclic voltammetry testing of a PC-based reference electrolyte, and another of the same composition but containing allura red was conducted (Fig. 5d). The results show that the cell containing allura red shows apparent larger anodic and cathodic absolute currents, which may be a sign of electrochemical activity in this region. However, as there are no new peaks appearing in the data coming from the cell containing allura red, it can be assumed that allura red does not undergo a faradaic reaction in itself within this voltage window. Also, note that the current in this test does not exceed 8 µA.

Considering the mild electrochemical effect of allura red within a battery environment, careful optimization of exposure time, layer thickness, and resin composition remains the preferred strategy to achieve a balance between print fidelity and electrochemical performance.

## Conclusions

This study demonstrates the successful formulation and 3D printing of GPEs for lithium-ion battery applications using a PEGDA-based photopolymerizable resin and carbonate-based liquid electrolytes. By tailoring the resin-to-electrolyte volume ratio, mechanically robust and ionically conductive GPEs were fabricated, achieving conductivities of 3.4 × 10^−3^ S cm^−1^ and 3.1 × 10^−3^ S cm^−1^ for DEC- and PC-based systems, respectively. The printed GPEs exhibited electrochemical stability up to ~4.7 V vs. Li^0^/Li^+^ and delivered performance comparable to tape-cast counterparts, confirming their viability for high-voltage cathodes. The 3D printing process provides precise geometric control and shape versatility while retaining good electrochemical performance even under ambient fabrication conditions. Symmetric cell cycling further confirmed stable operation and compatibility with lithium metal electrodes. These results highlight that 3D printed GPEs combine high ionic conductivity, electrochemical stability, and geometric tunability, establishing a scalable and adaptable platform for the design of next-generation, shape-conformable energy storage devices. Future efforts will focus on optimizing resin formulations without relying on UV absorbers to improve print fidelity, while preserving electrochemical properties. Additional directions include the incorporation of functional fillers to enhance mechanical strength or ionic conductivity, evaluation of alternative lithium salts and solvents to expand chemical compatibility, and integration of 3D printed GPEs into full-cell battery architectures. Such developments will further unlock the potential of additive manufacturing for advanced custom-shape lithium-ion batteries.

## Supplementary information

Supplementary Information

## Data Availability

The data that support the findings of this study are available from the corresponding author upon reasonable request.
