# Supplementary material for: Vat photopolymerization of gel polymer electrolytes with solvent-dependent performance and complex geometries for Li-ion batteries
Source: Commun Eng. 2026 May 5;5:128. doi: 10.1038/s44172-026-00682-9 (PMC13350908; doi:10.1038/s44172-026-00682-9)
Supplement: Supplementary file 1 — Supplementary Information [file 44172_2026_682_MOESM1_ESM.pdf]

Vat Photopolymerization of Gel Polymer Electrolytes with Solvent-Dependent Performance and Complex Geometries for Li-ion Batteries

Alexis Maurel[1]*, Katherine R. Gonzalez[1], Hugo A. Garcia[2], Laura C. Merrill[3], Ana C. Martinez[1,]*

[1] Metallurgy, Materials and Biomedical Engineering Department, The University of Texas at El Paso, El Paso, TX 79968, USA

[2] Department of Aerospace and Mechanical Engineering, The University of Texas at El Paso, El Paso, TX 79968, USA

[3] Sandia National Laboratories, Albuquerque, NM 87123, USA

Corresponding author: Alexis Maurel (amaurel@utep.edu)

Ana C. Martinez (acmartinezm@utep.edu)

Supplementary information

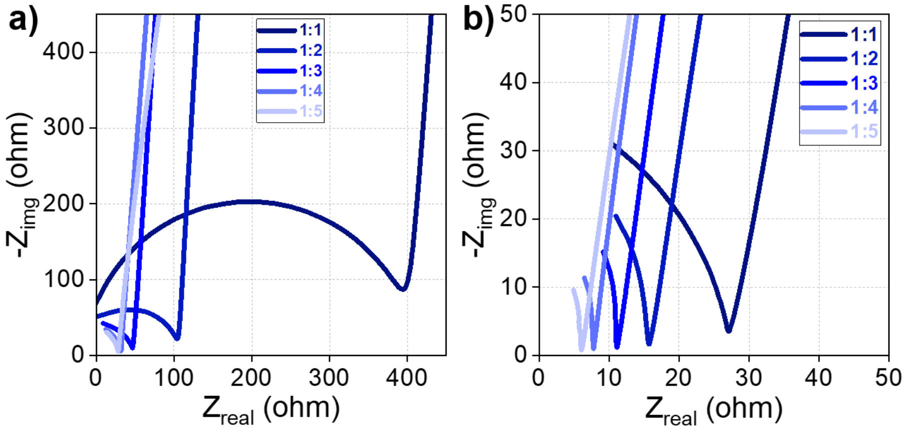

Figure S1. a) Representative Nyquist plots of the different tape cast ratios that were used to calculate the ionic conductivity data in **Figure 1a** and **1b**. **a)** DEC-based tape cast GPEs. **b)** PC-based tape cast GPEs. Note that the discs used for **(a)** had a diameter of 6.35 mm and those in **(b)** a diameter of 15.5 mm. The thicknesses were kept between 300-400 µm.
